# Severe immune thrombocytopenia that developed immediately after COVID-19 in a school-aged patient: A case report

**DOI:** 10.3389/fped.2023.1120093

**Published:** 2023-03-22

**Authors:** Kazuma Shinno, Yoshinori Banno, Isamu Kamimaki

**Affiliations:** Department of Pediatrics, National Hospital Organization Saitama Hospital, Saitama, Japan

**Keywords:** immune thrombocytopenia, SARS-CoV-2, COVID-19, viremia, children

## Abstract

Immune thrombocytopenia (ITP) is an autoimmune disorder that is sometimes triggered by a preceding viral infection and is characterized by a transient or persistent decrease in the platelet (Plt) count. Herein, we report the first pediatric case of severe ITP that developed immediately after the diagnosis of coronavirus disease 2019 (COVID-19) in a school-aged girl. A previously healthy six-year-old girl was diagnosed with COVID-19 a day before experiencing a high fever, sore throat, and headache. She also presented with gingival hemorrhage, petechiae around both eyes and on the chest, and ecchymosis on her right leg. Based on the mucosal hemorrhage and a very low Plt count of 3 × 10^3^/µl, we diagnosed her with severe ITP and urgently treated her with intravenous immunoglobulin (IVIG) to prevent life-threatening hemorrhage. The Plt count increased to 266 × 10^3^/µl one week after treatment with IVIG. Given the possibility of severe ITP secondary to COVID-19, patients with COVID-19 should be carefully examined for the signs of ITP, such as mucosal hemorrhage. Their Plt counts should also be monitored.

## 1. Introduction

Immune thrombocytopenia (ITP), which is relatively well-described in children, is an autoimmune disease characterized by idiopathic thrombocytopenia (1). ITP sometimes develops a day to a few weeks after a preceding viral infection, and severe ITP increases the risk of life-threatening hemorrhage (2). The coronavirus disease 2019 (COVID-19), caused by the severe acute respiratory syndrome coronavirus 2 (SARS-CoV-2), is likely to be mild or asymptomatic in most children. Among symptomatic cases, fever and cough are the major manifestations (3). Herein, we describe the first case of severe pediatric ITP that developed immediately after a COVID-19 diagnosis.

## 2. Case presentation

A previously healthy six-year-old girl, with no family history of autoimmune or hematologic diseases, developed purpura associated with one day of gingival hemorrhage. She also presented with fever, sore throat, headache, mild cough, and rhinorrhea on the same day. Her father had tested positive for SARS-CoV-2, based on reverse transcriptase-polymerase chain reaction (RT-PCR) two days prior, and her test result was likewise positive the day before she visited our hospital. Her anti-SARS-CoV-2 antibodies were not measured, as her SARS-CoV-2 RT-PCR test was positive. She had no preceding infection or vaccination within the previous month and was not taking any medications. She had never been vaccinated against SARS-CoV-2.

The patient's vital signs were as follows: body temperature, 40.2°C; pulse, 137 beats/min; respiratory rate, 32 breaths/min; and blood pressure, 115/55 mmHg. On physical examination, the patient looked pale but lively, was able to walk without any help, and spoke clearly. Her palpebral conjunctivas were not pale and had no hemorrhagic spots. She had gingival hemorrhage and redness of the pharynx without a white coat. On auscultation, her respiratory sounds were clear and without any rales bilaterally. She further presented with petechiae around both eyes, on her chest, and at the base of her neck and ecchymoses on her right leg.

**Figure 1:**
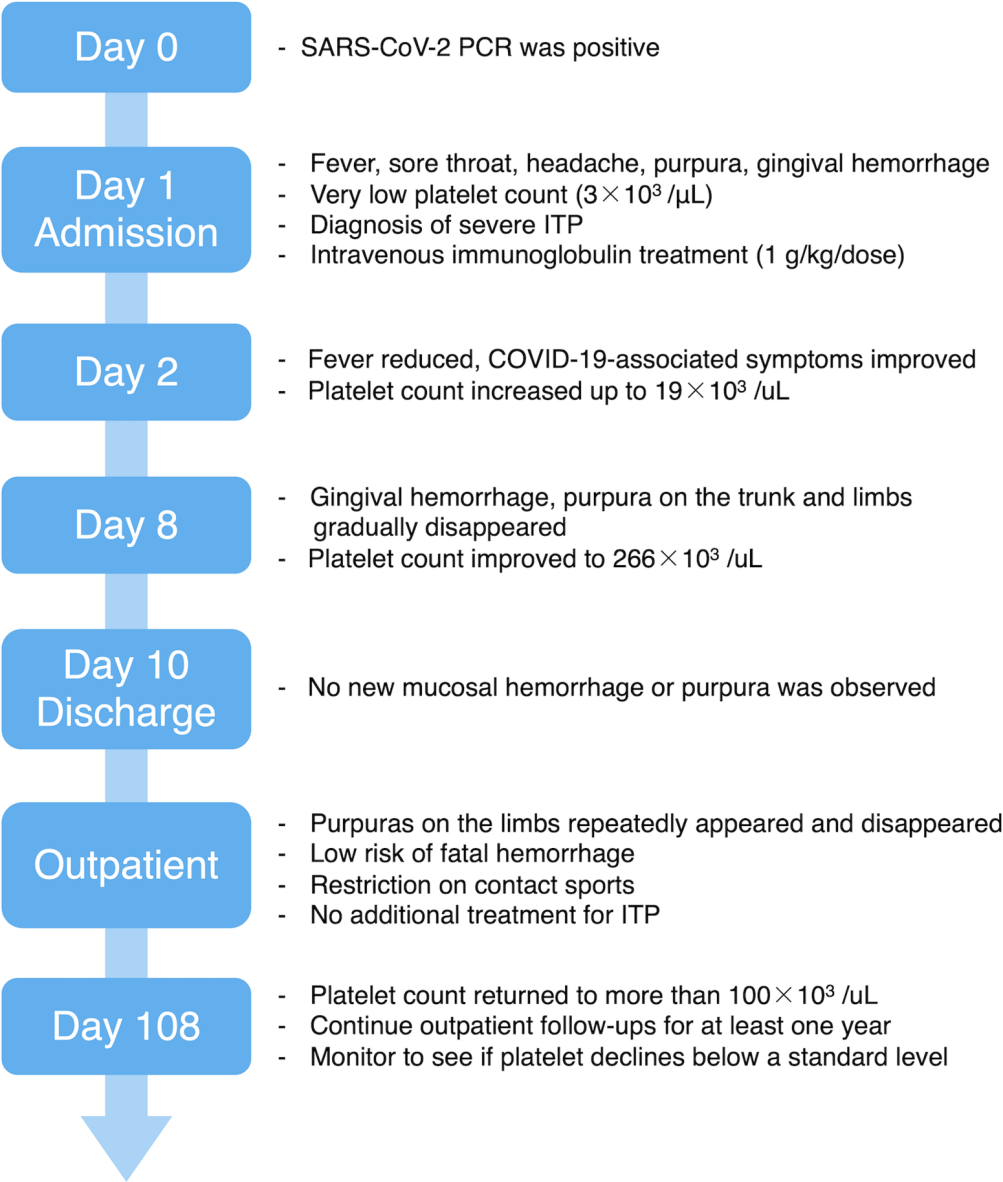
Timeline course of the patient.

Given that mucosal hemorrhage is a sign of life-threatening hemorrhage, she was hospitalized (day 1). On admission, she had a very low platelet (Plt) count (3 × 10^3^/µl), a normal white blood cell count [5.1 × 10^3^/µl (segmented cells 79%, lymphocytes 16%, monocytes 3%, eosinophils 1%, stab cells 1%)], and a normal hemoglobin level (12.5 g/dl). No blasts and no coagulation abnormalities were observed in her blood examination. Thus, we diagnosed her with severe ITP and treated her with 17.5 g (1 g/kg/dose) of intravenous immunoglobulin (IVIG). Due to her age, the absence of pneumonia, and no risk factors for severe COVID-19, she was not eligible for antivirals or monoclonal antibodies in Japan (4, 5). Therefore, we chose to closely monitor her COVID-19 symptoms without administering any treatments. The day after admission (day 2), her body temperature resolved to 37.1°C, and her cough, headache, sore throat, and rhinorrhea improved. Afterward, her respiratory symptoms did not worsen, and she did not require any respiratory support or oxygen administration. No new hemorrhagic spots emerged, and the existing purpura gradually disappeared. Her Plt count improved slightly on day 2 (19 × 10^3^/µl) and was within the normal range (266 × 10^3^/µl) on day 8. Both a rapid antigen test for group A beta-hemolytic streptococci and a stool antigen EIA test for *Helicobacter pylori* were negative. An antinuclear antibody test was non-reactive, and her Plt-associated IgG was within the normal reference range (Plt count: 24.3 ng/10^7^, day 17). No possible causes of ITP other than COVID-19 were identified. During hospitalization, the gingival hemorrhagic spots and purpura on the patient's trunk and limbs gradually disappeared. No new mucosal hemorrhage or purpura were observed, and the patient was discharged on day 10 (Figure 1).

During the outpatient follow-up, the patient's Plt count reduced to 25 × 10^3^/µl on day 24 and remained between 15 × 10^3^/µl and 32 × 10^3^/µl for the next two months. Purpura on the patient's lower legs appeared repeatedly and disappeared; however, no mucosal hemorrhage was observed. Owing to the low risk of life-threatening hemorrhage, we restricted the patient from contact sports and kept observing her without any additional treatment. On day 108, her Plt count improved to more than 100 × 10^3^/µl (121 × 10^3^/µl), but we did not suspend the follow-up procedure and planned to continue outpatient follow-ups for at least one year. We intend to carefully monitor her Plt count to see if it drops below a standard level, or if it turns into chronic ITP (Figure 1).

## 3. Discussion

We describe the first pediatric case of ITP that developed almost simultaneously with the onset of COVID-19. Moreover, the ITP was severe and was associated with mucosal hemorrhage and very low Plt levels (3 × 10^3^/µl).

To date, 13 pediatric cases of ITP secondary to COVID-19 have been reported (Table 1) (6–17). Among them, only one patient had developed ITP at almost the same time as the onset of COVID-19 (Case 1 in Table 1) (6). Furthermore, the patient developed ITP associated with COVID-19 twice, once on the day of the onset of COVID-19 (day 1) and again on day 25. The patient's Plt count on day 1 was 56 × 10^3^/µl, which improved without any treatment.

**Table 1:** Summary of the reported cases of pediatric ITP with COVID-19.

	Author	Age at diagnosis/sex	Duration from COVID-19 symptoms to ITP onset	Platelet count at diagnosis	Medical history
1	Soares et al *(*6)	2 years/female	(1st) the same day	56 × 10^3^/µl	Previously healthy
			(2nd) 25 days	28 × 10^3^/µl	
2	Tsao et al (7)	10 years/female	3 weeks	5 × 10^3^/µl	Previously healthy
3	Patel et al (8)	12 years/female	5 days	<10 × 10^3^/µl	Previously healthy, developed respiratory failure simultaneously
4	Ceglie et al (9)	11 years/male	4 weeks	5 × 10^3^/µl	Kawasaki disease (2 years old)
5	Behlivani et al (10)	15 years/male	5 weeks	1 × 10^3^/µl	Previously healthy
6	Behlivani et al (10)	3 years/female	3 weeks	26 × 10^3^/µl	Previously healthy
7	Rosenzweig et al (11)	16 years/male	3–4 weeks	45 × 10^3^/µl	Previously healthy
8	Dongre et al (12)	5.5 years/female	27 days	3 × 10^3^/µl	Acute lymphoblastic leukemia
9	Sivasankaran et al (13)	9 years/female	4 weeks	15 × 10^3^/µl	Previously healthy
10	Tariverdi et al (14)	7 years/male	No symptom. Possibly asymptomatic COVID-19 preceding ITP by 2 weeks.	8 × 10^3^/µl	Previously healthy
11	Vadakkekara et al (15)	1.5 years/female	2–3 weeks	20 × 10^3^/µl	Previously healthy
12	Aydin et al (16)	5 years/female	4–5 weeks	2 × 10^3^/µl	Previously healthy
13	Dominguez et al (17)	13 years/female	4 days	6 × 10^3^/µl	Previously healthy

Severe ITP is defined as clinically relevant hemorrhage necessitating treatment or a Plt count of 20 × 10^3^/µl or less (1, 18). The majority of COVID-19 patients with thrombocytopenia show a mild or moderate decrease in Plt count (>50 × 10^3^/µl) without hemorrhagic symptoms (19–21). However, if the Plt count is below 20 × 10^3^/µl or it reduces by >50% within 24–48 h, it is necessary to classify ITP as a differential diagnosis (21). In our case of the six-year-old girl, her Plt count was 3 × 10^3^/µl, and oral mucosal hemorrhage was also observed. Therefore, she was diagnosed with severe ITP with a high risk of life-threatening hemorrhage. In the guidelines from an expert group for adult ITP with COVID-19, IVIG is recommended when a rapid increase in the number of Plts is required due to bleeding (21). We decided to treat her with IVIG, instead of corticosteroids, to restore her Plt count quickly, and she responded to IVIG well. As noted above, the only previously reported patient who developed ITP almost simultaneously with the onset of COVID-19 did not have severe ITP at that time (6). To the best of our knowledge, our patient is the first pediatric case of severe ITP developing immediately after a COVID-19 diagnosis.

Regarding ITP associated with COVID-19, numerous potential mechanisms of hematopoietic dysfunction resulting from SARS-CoV-2 infection have been postulated. These mechanisms include: (1) the production of antibodies to megakaryocytes, and the initiation of apoptosis of their precursors due to viral infection of megakaryocytes; (2) the inhibition of differentiation and maturation of megakaryocytes by cytokines such as interleukin-1β, tumor necrosis factor-α and -β, and interferon-α; (3) the reduction in thrombopoietin production due to viral infection of hepatocytes; and (4) the decreased fragmentation of megakaryocytes and Plt production, and the increased consumption of Plts in pulmonary vessels due to viral lung damage (21, 22). However, no published paper has elucidated the timing of these proposed mechanisms after the onset of COVID-19. A review of the etiology of virus-associated ITP, not limited to COVID-19, presented two patterns based on the onset of viral infection: (1) ITP occurring during the viremic phase of acute virus illness and (2) ITP developing days to weeks following viral infection (2). During the viremic phase of an acute viral infection, it is hypothesized that Plt clearance depends on a viral antigen in the blood circulation (2). In our case, thrombocytopenia occurred almost simultaneously with the onset of COVID-19 and persisted for approximately three months, suggesting that both patterns could be functional.

In conclusion, we reported a pediatric case of severe ITP that developed simultaneously with COVID-19. COVID-19 could cause severe ITP with life-threatening hemorrhage immediately after its onset. Routine physical examination for purpura and mucosal hemorrhage should be performed in patients with COVID-19 to detect severe ITP earlier.

## Data Availability

The original contributions presented in the study are included in the article further inquiries can be directed to the corresponding author.
